# Water recovery of drying waste using a thermoelectric cooler and PV/T assisted

**DOI:** 10.1038/s41598-026-35137-w

**Published:** 2026-01-28

**Authors:** Amr Elbrashy, Magda K. El-fakharany, Maher Abou Al-Sood, F. Sh. Abou-Taleb, Fadl A. Essa

**Affiliations:** Mechanical Engineering Department, Faculty of Engineering, Kafrelsheikh University, Kafrelsheikh, 33516 Egypt

**Keywords:** Energy, Drying, PV/T, Thermoelectric, Water recovery, Sustainability, Energy science and technology, Engineering

## Abstract

Energy, food, and water are the most essential demands for the human community. In this study, a novel hybrid solar photovoltaic/thermal (PV/T) solar dryer integrated with a water recovery unit is designed, developed, and experimentally evaluated for drying agricultural products. The system combines a PV/T air collector that simultaneously generates electricity and hot air with a thermoelectric cooling (TEC) unit that condenses water vapor from the drying exhaust. This theoretical research aims to develop a new concept for drying systems based on thermoelectric coolers, using PV/T as a solar collector and an electric supplier. The scientific innovation lies in utilizing the waste heat from the hot side of the TEC units and introducing it into the drying chamber to enhance the evaporation rate. Meanwhile, the water vapor expelled through the cold side of the units is condensed, transforming the drying-to-drinking (D2D) process without energy or mass loss. Furthermore, drying 1 kg of tomato leads to two sources of condensed water (tomato moisture + atmospheric water). While an airflow rate of 0.05 kg/s corresponds to an inlet of atmospheric air at 1440 kg during 8 h, the water recovery unit produced nearly 3.9 L of water during an 8-hour drying cycle. Thus, addressing the dual challenge of food preservation and water scarcity.

## Introduction

Most industrial dryers use fossil fuels, which emit greenhouse gases and contribute to environmental pollution and global warming. Drying is very energy-intensive, needing between 14.53 and 90 MJ of energy to dry just 1 kg of food^1^. As fossil fuels become less available, more expensive, and environmentally harmful, there is an urgent need to develop drying systems that use clean and sustainable energy sources^2^. Solar energy is a prominent natural resource due to its widespread availability, easy access, and environmental benefits, as it doesn’t emit greenhouse gases^3^. Moreover, it can be exploited in various energy sources, including photovoltaic, thermal, and electric energy conversion systems^4,5^. Many solar systems, such as flat plates^6^, evacuated tubes^7,8^, and compound parabolic concentrators^9,10^ have been harnessed for solar drying applications. Besides these systems, photovoltaic/thermal (PV/T) collectors have gained growing interest due to their capability to produce both electricity and heat from solar radiation, boosting the overall efficiency of solar energy use^11,12^. Tiwari and Tiwari^13^ examined the exergy and economic performance of a hybrid PV/T solar dryer for greenhouse applications. The analysis uses both theoretical and experimental approaches to calculate parameters like energy and exergy efficiency, thermal energy, and embodied energy. Key findings include a significant improvement in drying efficiency, a short payback time of 1.23 years, and a life cycle assessment indicating lower environmental impacts. Gupta, et al.^14^ focused on developing a PV/T solar dryer to rely on renewable energy as much as possible. They found that the highest thermal efficiency of 29.17% occurred during sunny summer days, while the peak electrical efficiency of 10.14% was recorded in winter. The PV/T dryer reached an efficiency of 26.37%. Yüksel, et al.^15^ investigated a V-grooved double-pass PV/T solar dryer integrated with TES. Experimental results show enhanced thermal efficiency ranging from 40.94% to 77.18%, with additional improvements when TES is included.

Bayrak, et al.^16^ proposed and experimentally assessed a solar-assisted air-source heat pump drying system that integrates a flat-plate solar collector with a heat pump and a controlled drying chamber. Experimental investigations under different solar radiation and operating conditions demonstrated that the system could maintain higher drying air temperatures (40–55 °C) and better humidity control, leading to improved product quality and reduced drying time. The system achieved a coefficient of performance (COP) between 3 and 4.2 representing a 25–35% energy saving compared to conventional heat pump drying. Hao, et al.^17^ experimentally tested a hybrid PV/T-SD to evaluate its drying efficiency, thermal performance, and environmental impact compared to direct and mixed-mode solar dryers with Lemon slices as a test material, integrating life cycle assessment. Results showed that the mixed-mode achieved the highest thermal efficiency 46.76%, whereas the PV/T-SD exhibited the highest overall efficiency 60.97%. Through the integration between PV/T and TEC, Vitulli, et al.^18^ tested across three climates (Macapá, Almería, and Wien) to assess the adaptability of electrical performance. Results showed that electricity production increased by up to 5.8%, 6.6%, and 3.5% in the respective locations, with corresponding gains in electrical and exergy efficiency. However, when thermal energy was included, PV/T-TEC performance became less favorable than PV/T alone. Nighttime electricity generation was achieved, but was negligible (0.017–0.044 W). Almería was identified as the most suitable site due to favorable climate conditions, while higher flow rates improved system efficiency but reduced TEC output. TEC can be used as a desalination application in different ways. Atmanandmaya, et al.^19^ proposed a hybrid-source thermal desalination system that integrates TEC modules to improve energy efficiency and water productivity. Experimental results show a 50% increase in freshwater production compared to conventional heater-only systems, significantly reducing condensation onset time. The system operates with high COP values (up to 8.30) and is adaptable to renewable energy sources, making it suitable for off-grid applications. Poblete and Bakit^20^ evaluated the effectiveness of humidification-dehumidification systems for recovering water from industrial sludge, specifically from the scallop aquaculture industry. Different condensation systems, including heat exchangers and TEC modules, were compared. The heat exchanger systems yielded higher water recovery rates, and although TEC modules showed lower efficiency, they proved to be cost-effective for small-scale applications. Congedo, et al.^21^ proposed a device combining TEC cells and an earth-to-air heat exchanger to extract water from humid air. The system is designed for developing countries and powered by renewable sources like photovoltaic panels and micro wind turbines. The system’s low cost, ease of installation, and portability make it suitable for off-grid applications in areas facing water scarcity. Application of TECs to air cooling and dehumidification has been extensively studied. This includes an investigation into TEC-driven humidification-dehumidification (HDH) systems for water desalination. Ashour, et al.^22^ experimentally analyzed an HDH system with an evaporative pad and TEC units to obtain the highest water production. They followed the approach of analyzing performance at various air speeds and TEC voltages. The principal conclusion was that optimum performance was gained with a gain-output ratio of 1.374 and COP of 0.78 at low air speeds (0.5–1.0 m/s) where residence time for condensation was sufficient. This presents a major compromise between airflow and condensation performance, a critical open-loop design parameter in the present investigation. Another relevant study by Han, et al.^23^ comprised an experimental examination of a closed-air circulation domestic water purification system. Based on the HDH principle, the device was driven by a TEC to offer an efficient alternative to conventional purification technology. The study employed a methodology that entailed designing a closed-loop system intended to reclaim the thermal energy from the hot side of the TEC to advance the humidification process. The key findings revealed high sensitivity of system operation to the water temperature at the inlet; most notably, the production rate of pure water and the output per unit of electric power consumption were at optimal levels of 414 g/h and 401 g/(kW·h) at an inlet temperature of 5 °C. A particular area of research that is directly in line with the objectives of the present study is the use of TECs for air cooling and dehumidification. TEC-based atmospheric water extraction, sharing principles with a closed-loop dehumidification cycle, has been comprehensively reviewed by Zheng, et al.^24^. Their research explains that these systems’ performance greatly relies on the physical geometry of the hot and cold junctions and operating conditions like input current and air velocity. The key findings are that cold-end fin geometry optimization and effective hot-end heat dissipation mechanisms are critical methods of optimizing water production efficiency. This underlines the need for effective thermal management, a conclusion which is borne out by Meng, et al.^25^, whose investigation of air-cooled thermoelectric generators determined that air-side heat sink thermal resistance was the primary performance-limiting factor. Continuing along this line of inquiry, Dizaji, et al.^26^ investigated a new, energy-free heatsink side cooling technique. They aimed to augment power generation with increased temperature difference. They greatly improved performance through the use of an ultrathin hydrophilic self-wicking sheet in creating a thin continuous water film for evaporative cooling. Their results indicated that the passive cooling method yielded a 50–150% increase in power output compared to conventional fan-heatsink methods, particularly under extreme ambient temperatures. Aside from external thermal management, internal thermoelectric module geometry is also a field that can be optimized. Table 1 summarizes the above literature, highlighting the key findings of each.

Most previous studies on the use of TEC systems focus on increasing moisture removal efficiency or improving energy consumption, without considering the utilization of waste heat or the potential for water recovery resulting from the drying process. Furthermore, employing a multi-TEC unit to perform two complementary functions has not yet been addressed. This opens a new area of research to improve the combined energy and water efficiency of drying systems.

The main contribution of this research is the introduction of an innovative concept, “From Drying to Drinking (D2D),” based on the design of a low-temperature drying system that maximizes the utilization of both the heat energy and water generated during the process. This design combines preserving the quality of the dried product, reducing energy consumption, and enhancing the sustainability of water resources through the recovery of waste water. The research also presents an integrated mathematical model to estimate the thermal and mass performance of the system, paving the way for future practical applications in the fields of food preservation, water security, and clean energy technology. This comprehensive design represents an important step towards zero-waste solar energy, contributing to clean production, resource efficiency, and sustainable food processing technologies.

**Table 1:** Summary of literature.

Authors/Refs.	System type / focus	Key parameters evaluated	Major findings / results
Tiwari & Tiwari^13^	Hybrid PV/T solar dryer for greenhouse	Energy & exergy efficiency, thermal & embodied energy, life cycle cost	Drying efficiency improved significantly; payback period = 1.23 years; reduced environmental impacts
Gupta et al.^14^	PV/T solar dryer relying on renewable energy	Thermal & electrical efficiency	Max thermal efficiency 29.17% (summer); max electrical efficiency 10.14% (winter); overall 26.37%
Yüksel et al.^15^	V-grooved double-pass PV/T dryer with TES	Thermal efficiency, storage effect	Thermal efficiency 40.94–77.18%; TES improved stability and energy utilization
Bayrak et al.^16^	Solar-assisted air-source heat pump dryer	COP, drying temp, humidity control	COP 3–4.2; 25–35% energy saving; better quality & reduced drying time
Hao et al.^17^	Hybrid PV/T solar dryer (Lemon slices)	Thermal & overall efficiency, environmental impact	Mixed-mode dryer: 46.76% thermal efficiency; PV/T-SD: 60.97% overall efficiency
Vitulli et al.^18^	PV/T–TEC hybrid under three climates	Electrical & exergy efficiency, climate adaptability	Electricity output ↑5.8–6.6%; night generation negligible; Almería optimal site
Atmanandmaya et al.^19^	TEC-assisted hybrid thermal desalination	COP, freshwater production	50% more freshwater vs. conventional; COP up to 8.3; faster condensation
Poblete & Bakit^20^	HDH system for industrial sludge water recovery	Water recovery rate, cost analysis	Heat exchangers > TEC in efficiency; TEC is cost-effective for small-scale
Congedo et al.^21^	TEC + Earth-to-air heat exchanger for water extraction	Water yield, system adaptability	Low-cost, portable system; suitable for off-grid & developing regions
Ashour et al.^22^	TEC-driven HDH system with evaporative pad	Air speed, voltage, COP, GOR	Optimum: GOR 1.374, COP 0.78 at 0.5–1.0 m/s; trade-off between airflow & condensation
Han et al.^23^	Closed-air loop TEC-based water purifier	Water temp sensitivity, production rate	Optimum: 414 g/h water, 401 g/kWh at 5 °C inlet
Zheng et al.^24^	Review of TEC-based atmospheric water extraction	Design geometry, heat dissipation, current & velocity	Fin geometry & hot-side cooling critical for water yield
Meng et al.^25^	Air-cooled thermoelectric generator	Heat sink thermal resistance	Air-side thermal resistance = main performance limiter
Dizaji et al.^26^	Energy-free cooling for TEC/TEG	Evaporative cooling film, temperature difference	50–150% ↑ in power vs. fan cooling; better under high temp

## Methodology

**Fig. 1:**
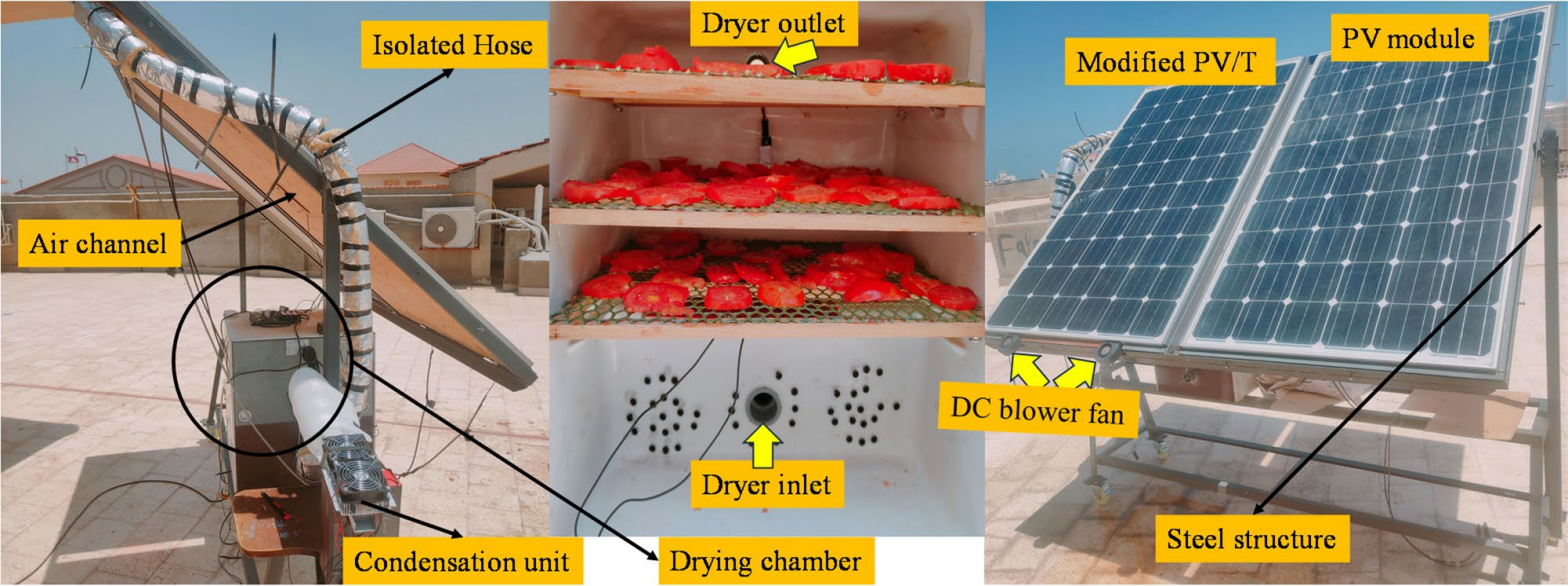
Experimental setup of the proposed hybrid PV/T combined drying chamber and condensation unit.

**Fig. 2:**
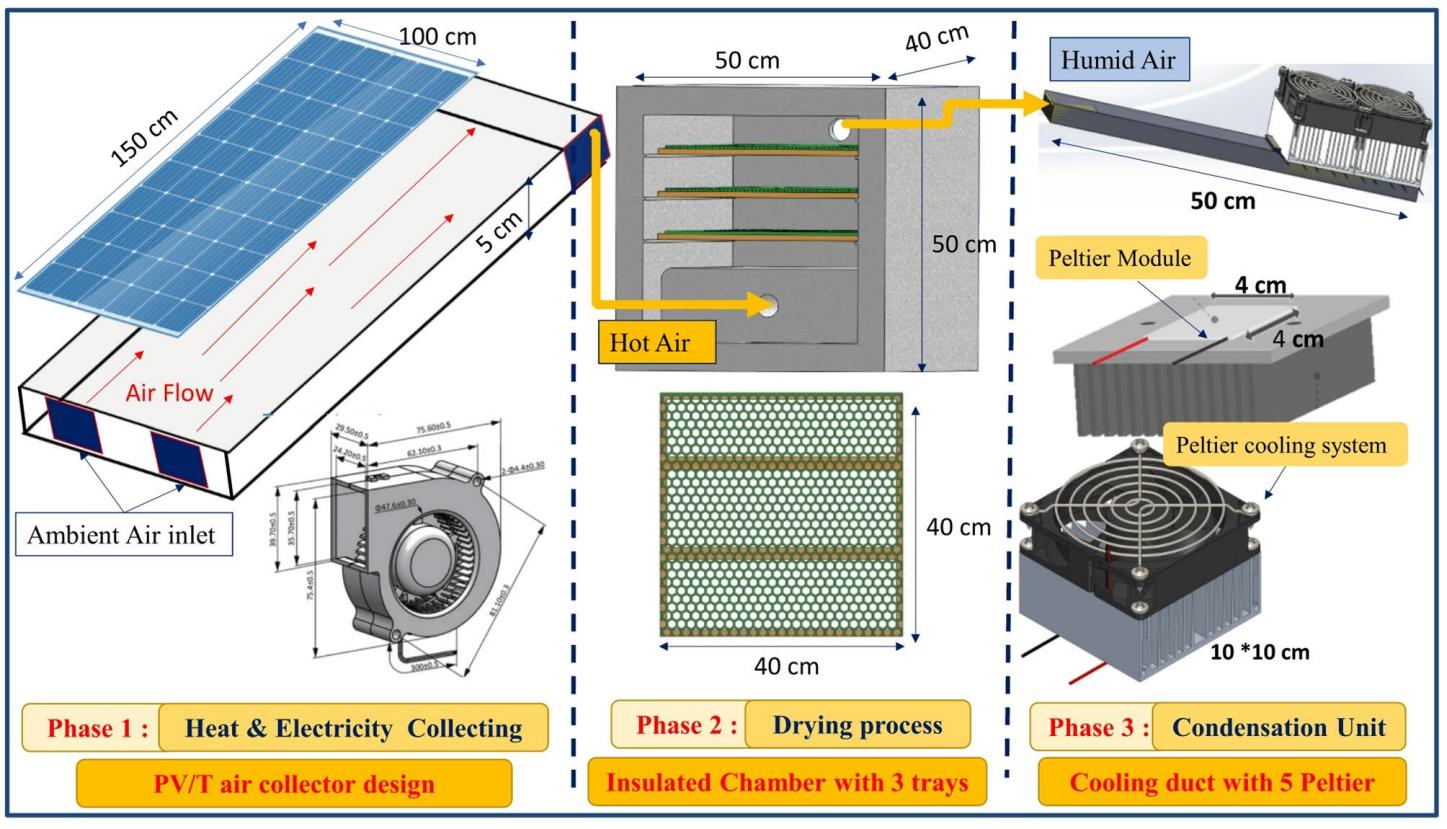
Schematic representation of the system’s operating and dimensions of the instruments.

The proposed system innovatively combines four key components, offering multiple solar-powered functions at once. These include PV/T cooling via forced air to improve electrical efficiency and prevent thermal stress, drying fruits with heated air, and water extraction by condensing moisture from wet air using TEC. The experimental setup photograph in Fig. 1 illustrates the whole system’s main components, representing each part. The detailed configuration and dimensions of the combined system are demonstrated schematically in Fig. 2. The system functions autonomously, with the blower fan powered by DC electricity generated from two PV panels, each with a capacity of 280 W. The experimental work took place in New Damietta City, Egypt, on the Mediterranean coast. Geographically, the site lies at latitude 31.4213° N and longitude 31.8144° E, which is characterized by moderate solar radiation levels, relatively high humidity, and seasonal temperature variations. The next subsection provides a detailed overview of each system component and the measuring devices used.

### Phase 1: PV/T air collector

The proposed system contains two PV solar panels, each consisting of 60 cells in a 6 × 10 configuration, with each cell sized at 156 mm × 156 mm. It provides a rated power up to 295 W, an open-circuit voltage up to 39.7 V, and an electrical conversion efficiency ranging from16.5% to 18.0% under standard test conditions. The panel measures 1500 × 1000 × 35 mm, and a weight of 18.6 kg. A 5 cm-deep air channel, having a hydraulic diameter of 9.5 cm for each inlet fan, is integrated into the panel and built from wood, with thermal insulation provided by wood-based materials below. Foam glass wool insulates the air hoses linking to the drying chamber to reduce heat loss during hot air transport. The setup incorporates four 15 W DC blower fans running at 12 V. Two fans are located at the air channel inlet, and one at the channel outlet to pull air and direct it into the drying chamber. Air passes through a 5 cm-deep channel under the PV panel, serving as a heat exchange area between the panel’s heated surface and the flowing air.

### Phase 2: drying chamber

The drying chamber measures 40 × 40 × 40 cm internally and is fully thermally insulated. It features three vertically stacked trays, each 40 × 40 cm with a thickness of 0.5 cm, spaced 7 cm apart to promote even hot air distribution and ensure thorough drying of plant materials. The trays are constructed from high-density polyethylene plastic mesh, chosen for its high resistance to heat from solar drying and food safety. The mesh has hexagonal openings with a 1 cm diameter and 0.25 cm thickness, allowing efficient hot air flow and improving heat and mass transfer. Reinforced with a heat-treated beech wood frame, the mesh provides necessary support to hold fruits and moist materials securely.

### Phase 3: condensation unit

The condensation channel was constructed using five TEC modules, each with dimensions of 4 × 4 cm. The cold side of the TEC modules was positioned in the path of the wet air, while the hot side (heat-dissipation side) was equipped with two cooling fans to enhance TEC module efficiency and dissipate heat quickly, preventing damage to the modules. The dimensions of the one fan are 10 × 10 cm, totaling 20 cm, equal to 5 TEC length. An aluminum heat sink with dimensions 10 × 10 cm has been integrated into the airflow path to further enhance the cooling efficiency by increasing the surface area for heat exchange, thus ensuring that the heat from the hot side of the TEC modules is rapidly dissipated. This is crucial in preventing the TEC modules from overheating, as excessive heat buildup could reduce their effectiveness and potentially lead to module failure.

### Measurement sensors

The temperature was measured with an FY-10 digital thermocouple sensor featuring a digital LCD display and a moisture- and water-resistant probe, ensuring accurate readings in a high-humidity drying setting. The sensor has an operating range of -50 to 110 °C and a measurement accuracy of ± 0.1 °C, making it appropriate for monitoring temperatures at various system locations, including the air duct, the rear of the PV panel, and the drying chamber. Relative humidity at the inlet and outlet of the drying chamber was determined using a DHT11 sensor, which provides reliable temperature and humidity data to monitor air condition changes throughout the drying process. Air velocity was assessed with a UT363S digital anemometer, offering a range of 0.5 to 30 m/s and an accuracy of ± 5%, allowing for the evaluation of fan performance and airflow efficiency across different stages of the system. Solar radiation intensity was quantified using an SM206 digital solar radiation meter with a broad range of 0–4000 W/m² and an accuracy of ± 5%. Table 2 shows the total uncertainty matrix data for these instruments and derived parameters, used to calculate the combined thermal efficiency uncertainty according to^27^:

1 $$\:{u}_{R}=\sqrt{\sum\:_{i=1}^{n}\:\:{\left(\frac{\partial\:R}{\partial\:{x}_{i}}{u}_{{x}_{i}}\right)}^{2}}\:$$

where a parameter $\:R=f\left({x}_{1},{x}_{2},\dots\:,{x}_{n}\right)$, the total uncertainty $\:{u}_{R}$ is calculated using the root sum of squares method^28^:

**Table 2:** The overall uncertainty of the measuring sensors.

Measurement	Parameter	Overall uncertainty
UT363S Anemometer	velocity (m/s)	$\:\sqrt{{0.5}^{2}+{0.3}^{2}}\approx\:0.58$ m/s
FY-10 Thermocouple	Temperature (ºC)	$\:\sqrt{{0.1}^{2}+{0.3}^{2}}\approx\:0.3$ ºC
SM206 Solar Meter	Radiation (W/m^2^)	$\:\sqrt{{10}^{2}+{0.1}^{2}}\approx\:10$ W/m^2^
Digital Balance	Mass loss (kg)	$\:\sqrt{{0.02}^{2}+{0.01}^{2}}\approx\:0.02$ kg
DHT11 Sensor	Relative humidity (%)	$\:\sqrt{{5}^{2}+{1}^{2}}\approx\:5$ %

### Theoretical framework

PV/T solar dryer transfers heat to the moist product via convective hot air, causing moisture to evaporate and be carried away by airflow.

The dry basis moisture content is defined as the ratio of the mass of water present in the product to the mass of dry solids. This parameter is commonly adopted in drying studies because it remains independent of the moisture level and provides a reliable basis for mass transfer analysis.

2 $$\:{X}_{db}\left(t\right)=\frac{{m}_{w}\left(t\right)}{{m}_{ds}}$$

The wet basis moisture content expresses the fraction of water relative to the total mass of the material (water + dry matter). Although less commonly used in modeling, it is widely reported in food science literature due to its intuitive physical meaning for practical applications.

3 $$\:{X}_{wb}\left(t\right)=\frac{{m}_{w}\left(t\right)}{{m}_{ds}+{m}_{w}\left(t\right)}\times\:100$$

The moisture ratio is a dimensionless parameter that normalizes the instantaneous moisture content against the initial and equilibrium moisture levels. This form facilitates the development of drying kinetics models and allows comparison between different drying conditions and products.

4 $$\:MR\left(t\right)=\frac{{X}_{db}\left(t\right)-{X}_{eq}}{{X}_{db,0}-{X}_{eq}}$$

The variation of moisture content with time is often described using a first-order exponential model, which assumes that the drying rate is proportional to the difference between the instantaneous and equilibrium moisture levels. This simplified empirical expression has been widely applied in thin-layer drying studies.

5 $$\:{X}_{db}\left(t\right)={X}_{eq}+\left({X}_{db,0}-{X}_{eq}\right){e}^{-kt}$$

The instantaneous mass of the product during drying is determined by summing the mass of dry matter (constant throughout the process) and the residual water content. This relation provides a direct link between moisture dynamics and the actual sample weight.

6 $$\:m\left(t\right)={m}_{ds}+{X}_{db}\left(t\right)\cdot\:{m}_{ds}$$

The incremental amount of water removed in a given time step can be calculated as the difference between the water content at consecutive intervals. This value is essential for quantifying drying performance and assessing energy requirements.

7 $$\:{m}_{w}\left(t\right)={X}_{db}\left(t\right)\cdot\:{m}_{ds}$$

8 $$\:{\Delta\:}{m}_{w}\left(t\right)={m}_{w}(t-{\Delta\:}t)-{m}_{w}\left(t\right)$$

Drying efficiency quantifies the fraction of supplied heat that is effectively utilized in moisture evaporation. It is calculated as the ratio of the energy associated with water phase change to the total heat input, providing an overall indicator of the thermal performance of the drying system.

9 $$\:\eta\:\left(t\right)=\frac{{m}_{\mathrm{evap\:}}\left(t\right)\cdot\:{h}_{fg}}{{Q}_{\mathrm{in\:}}}\times\:100$$

The total cooling load on the thermoelectric cooler’s cold side is represented below. It accounts for both sensible cooling of the moist air stream and latent cooling due to condensation of water vapor, expressed on a dry-air basis. It is fundamental for coupling psychrometric processes with thermoelectric cooling performance.

The cooling capacity of a thermoelectric module can be determined using its physical parameters, as demonstrated in Table 3. The first term represents the Peltier effect (useful cooling), the second accounts for internal Joule heating, and the third denotes parasitic back-conduction of heat from the hot side. It provides the theoretical performance of the TEC at given current and junction temperatures^19^.

10 $$\:{Q}_{c}=\alpha\:I{T}_{cold}-\frac{1}{2}{I}^{2}R-K\left({T}_{hot}-{T}_{cold}\right)$$

The total heat must be dissipated from the hot side of the thermoelectric module. It is equal to the cold-side load plus the electrical input power to the device, consisting of resistive losses and the additional Peltier heating. This relation is critical for sizing the heat sink and maintaining thermal stability of the module.

11 $$\:{Q}_{h}={Q}_{rej}={Q}_{c}+{P}_{TEC}$$

12 $$\:{P}_{TEC}={I}^{2}R+\alpha\:I{\Delta\:}T$$

The rate of condensate water collected from the drying air stream. It is based on the reduction of the humidity ratio across the thermoelectric dehumidifier, directly linking system performance to water recovery potential^29^.

13 $$\:{\stackrel{\prime }{m}}_{cond}={\stackrel{\prime }{m}}_{air}\left({\omega\:}_{in}-{\omega\:}_{out}\right)$$

The COP measures the efficiency of the thermoelectric cooling process by relating useful cooling capacity to the electrical power consumed. It is an essential figure of merit for evaluating the energy effectiveness of TEC-assisted drying and water recovery systems^19^.

14 $$\:COP=\frac{{Q}_{cold}}{{P}_{elec}}=\frac{\alpha\:I{T}_{cold}-\frac{1}{2}{I}^{2}R-K{\Delta\:}T}{{I}^{2}R+\alpha\:I{\Delta\:}T}$$

**Table 3:** TEC performance specifications.

Parameter	Symbol	Value	Units
Maximum current	I_max_	6.0–6.4	Amp
Maximum voltage	V_max_	14.0–15.4	Volt
Maximum power input	P_max_	80–90	Watt
Maximum temperature difference	ΔT_max_	75	°C
Maximum cooling capacity	Q_max_	50–60	Watt
Seebeck coefficient (total)	α	0.05	V/K
Electrical resistance	R	1.9	Ω
Thermal conductance	K	0.5	W/K
Module dimensions	–	40 × 40 × 4	mm

## Results and discussion

The proposed system represents an integrated PV/T air collector designed to simultaneously generate power and dry agricultural produce, demonstrating an advanced application of hybrid renewable energy technology.

### Psychometric processes

**Fig. 3:**
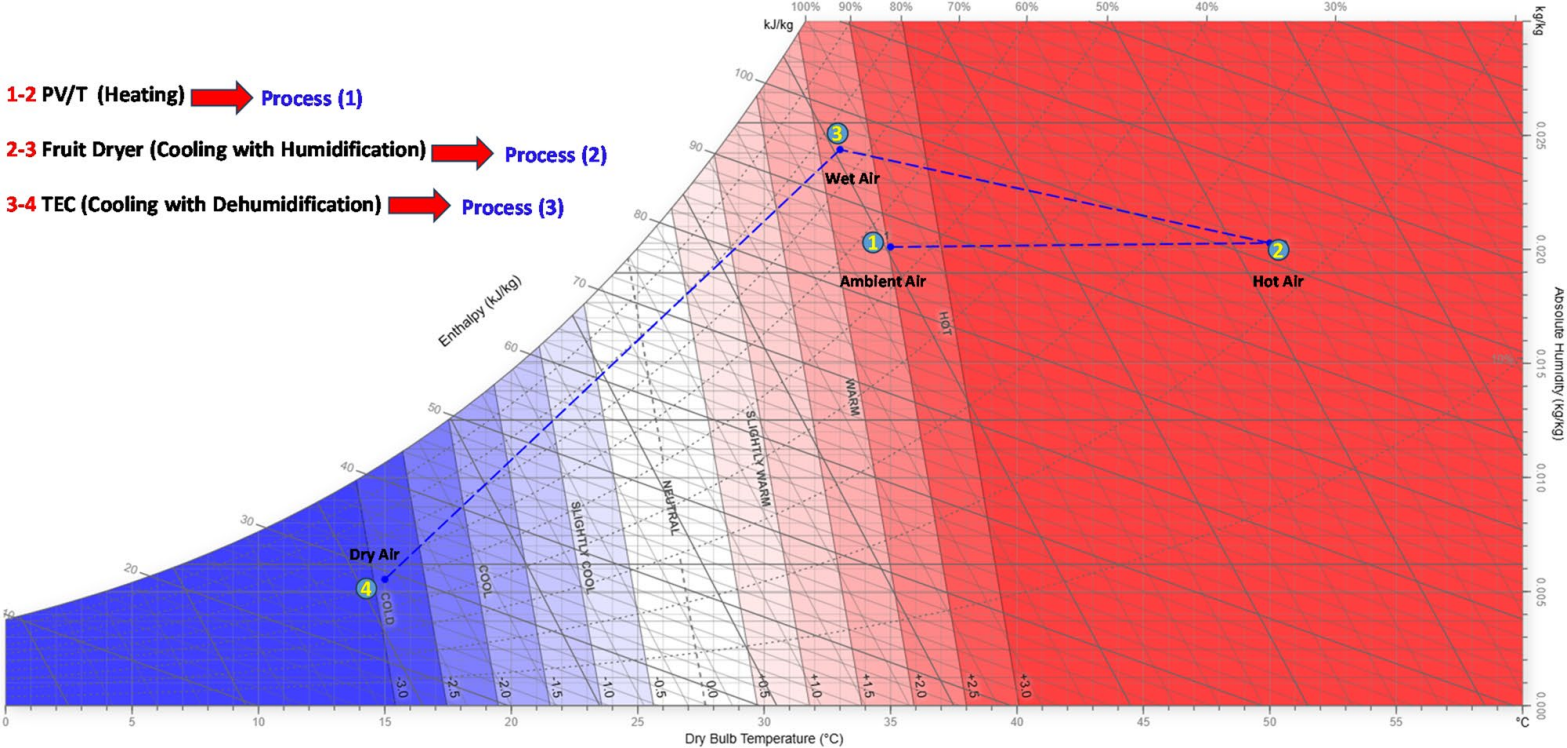
Psychometric processes of the system behavior.

The psychrometric chart in Fig. 3 illustrates the thermodynamic pathway of air during heating, cooling, humidification and dehumidification in the proposed system. The average data of each state have been represented to demonstrate the thermodynamic pathway of air. Firstly, ambient air (point 1) enters at a dry-bulb temperature (DBT) of approximately 35 °C and a relative humidity (RH) of 55%, corresponding to an absolute humidity ratio (ω) of about 0.025 kg water/kg dry air. After heating (point 2), the air temperature rises to nearly 52 °C, while the humidity ratio remains constant, indicating a sensible heating process. Subsequent cooling (point 3) brings the air toward the saturation curve, and further cooling below the dew point (point 4) reduces the humidity ratio to approximately 0.005 kg H₂O/kg dry air. The net change in humidity ratio (Δω ≈ 0.020 kg H₂O/kg dry air). So, for each kg of dry air processed, about 20 g of water can be extracted by TEC cooling, depending on the type of dried material.

### PV/T evaluation

**Fig. 4:**
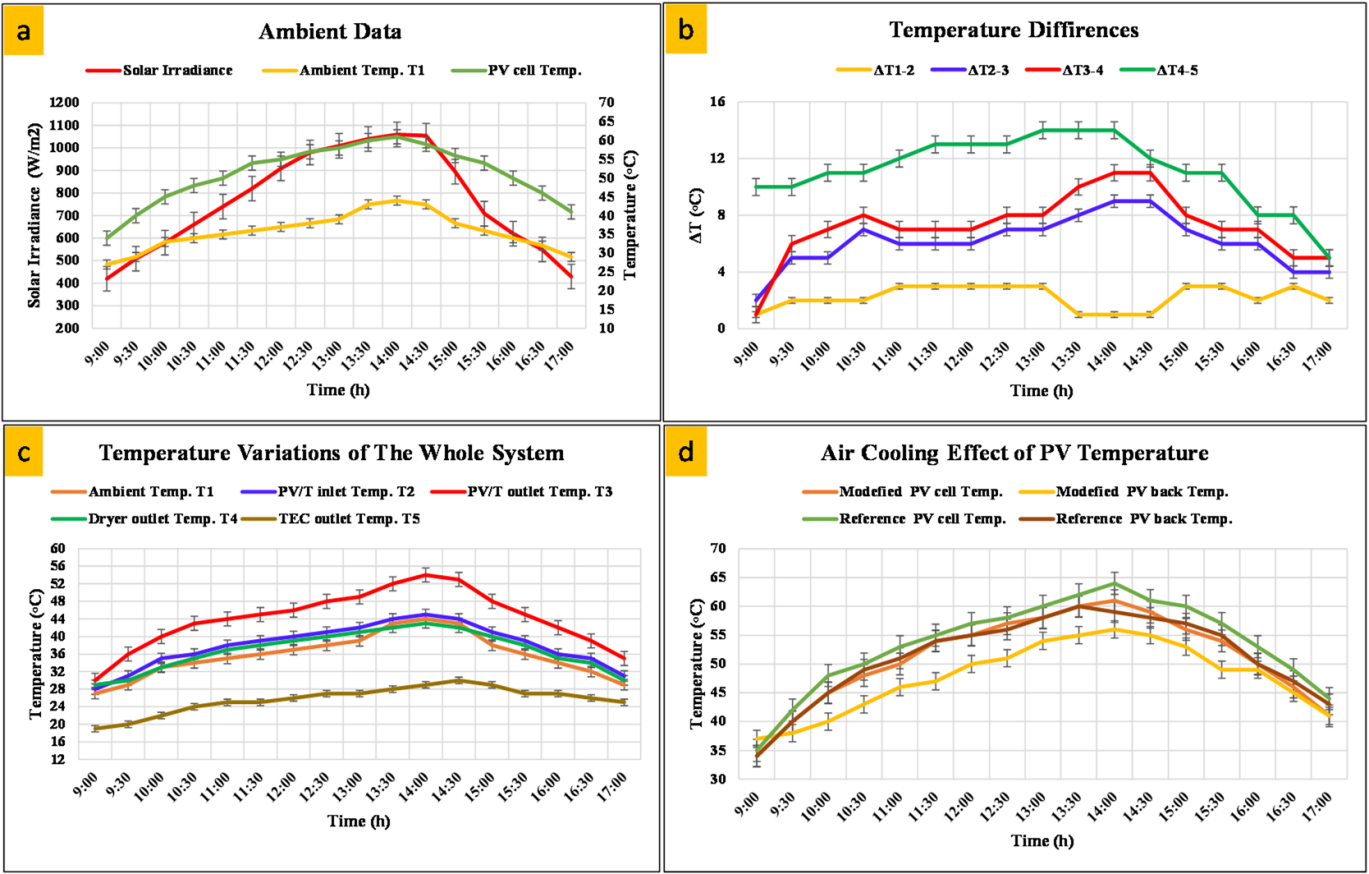
(**a**) Influence of PV cell temperature by ambient variation, (**b**) Temperature rise between each system’s point, (**c**) Temperature trend of the whole system, and (**d**) comparison between cooled and uncooled PV module.

Figure 4 shows the ambient temperature variations affecting the PV/T system. Figure 4a shows the ambient temperature rising gradually with the increase in solar radiation starting from 9:00 until 14:30. The top surface (glazed) of the PV/T heats up gradually until it reaches a maximum temperature of 60 °C at 14:00. It is worth mentioning that the top surface temperature of the PV/T also fluctuates with wind speed, as convective heat transfer affects the surface heating. Figure 4b shows the temperature rise between each point in the system, where ΔT1-2 represents the difference between the inlet temperature and the ambient temperature, which does not exceed 2 °C at most. This rise of one or two degrees is due to the heating of the fan rotor during continuous operation. ΔT2-3 represents the temperature rise between the air outlet of the solar cells and the inlet, which reaches a maximum value of 11 °C due to turbulent flow and enhanced heat transfer in the iron mesh. ΔT3-4 value represents the temperature difference between the outlet of the drying chamber and the outlet of the PV/T, which is relatively small due to the product’s heat absorption and the chamber’s walls. ΔT4-5 refers to the temperature difference between the outlet of the dryer and the cooling unit (TEC) outlet, through which the water is condensed. The overall temperature variations of the entire system are synthesized in Fig. 4c to provide a wide understanding of how the system temperature behaves. The trajectory of each temperature node physically narrates the energy flow. Ambient air (T1) is drawn into the system and heated as it passes through the PV/T, exiting as T2. It is further heated through the solar radiation and conduction heat transfer between PV/T layers and by forced internal convection to become T3. This now-hot air enters the drying chamber, transferring its latent and sensible heat to the moist product, resulting in a slight temperature drop at the dryer outlet (T4). Finally, the air passes over the cold side of the TEC, where it is actively cooled to temperature T5 before potentially being exhausted. The profound impact of the active air-cooling mechanism on the modified (cooled) and reference (uncooled) PV modules is demonstrated in Fig. 4d. The reference PV cell temperature rises significantly, closely tracking solar irradiance and reaching peak values of 65 °C. In contrast, the modified air-cooled PV module temperature drops significantly, by about 5 °C. This reduction is achieved by transferring the convection from the cells to the air-flowing coolant.

### Energy and exergy evaluation

**Fig. 5:**
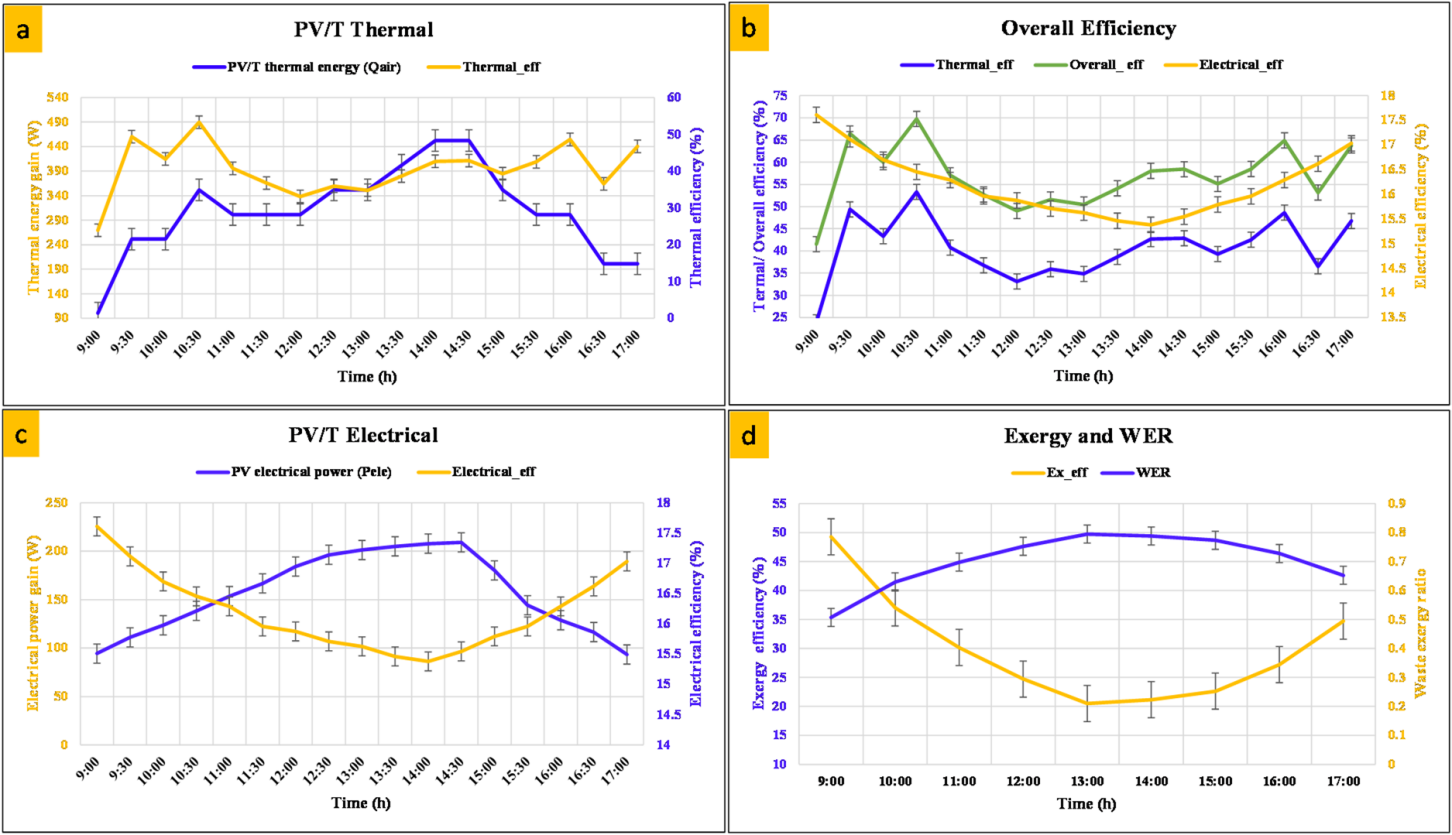
Performance characteristics of the PV/T hybrid system over a daily operating period: (**a**) thermally, (**b**) overall efficiency, (**c**) electrically, and (**d**) exergy efficiency and Exergy Analysis as a function of time.

The complementary behavior of thermal and electrical efficiencies underscores the advantage of integrated energy and water recovery in enhancing the sustainability of agricultural drying applications. Figure 5 illustrates the dynamic performance of the PV/T system while drying one kilogram of tomatoes at an average drying temperature of 40 °C throughout the day. As shown in Fig. 7a, the thermal energy gain gradually increased starting in the morning hours, peaking around midday when solar radiation peaked, before declining as the evening approached. This trend directly impacted thermal efficiency, exhibiting fluctuations between 35% and 60%, highlighting the sensitivity of convective heat transfer to changes in solar radiation intensity and ambient conditions. In Fig. 5b, the overall efficiency profile shows the combined contribution of both thermal and electrical efficiencies. While the electrical efficiency remained relatively stable (14%–17%), the greater variability in thermal efficiency resulted in the overall efficiency peaking during the mid-morning and late afternoon. The electrical performance, shown in Fig. 5c, shows that the solar module generated maximum power (over 200 W) during midday when irradiance was at its peak. However, the corresponding electrical efficiency decreased slightly at high temperatures due to the PV cells’ known negative temperature coefficient. This observation underscores the importance of efficient heat extraction from the PV cell surface to mitigate electrical losses. Figure 5d shows the thermal exergy efficiency and WER. The thermal exergy efficiency remained within 40–55% range, peaking at noon, indicating that the system effectively converted available solar energy into useful work under high solar intensity.

In PV/T systems, the efficiency is usually separated into thermal, electrical, and an overall efficiency that accounts for both. Thermal efficiency represents the fraction of incident solar energy converted into useful heat by the thermal collector and can be calculated by^30^:

15 $$\:{\eta\:}_{thermal}=\:\frac{{\stackrel{\prime }{m}}_{air}{C}_{p,air}\left({T}_{out}-{T}_{in}\right)}{G\cdot\:{A}_{s}}$$

While electrical efficiency represents the fraction of solar radiation converted into electrical power by the PV module, it depends on temperature^31^.

16 $$\:{\eta\:}_{elec}={\eta\:}_{ref}\left[1-{\beta\:}_{temp}\left({T}_{PV}-25\right)\right]$$

Overall efficiency represents the total fraction of incident solar energy converted into both electricity and heat^30^:

17 $$\:{\eta\:}_{total}={\eta\:}_{thermal}+{\eta\:}_{elec}$$

The thermal exergy carried by the heated air exiting the PV/T channel is calculated using the following expression^32,33^:

18 $$\:E{x}_{\mathrm{th\:}}={\dot{m}}_{\mathrm{air\:}}{C}_{p}\left[\left({T}_{\mathrm{out\:}}-{T}_{\mathrm{amb\:}}\right)-{T}_{\mathrm{amb\:}}\mathrm{ln}\left(\frac{{T}_{\mathrm{out\:}}}{{T}_{\mathrm{amb\:}}}\right)\right]$$

Where, $\:E{x}_{\mathrm{th\:}}$is Thermal exergy (W) and $\:{T}_{\mathrm{amb\:}}$is Ambient temperature (K).

Assuming the electrical energy produced by the PV module is entirely usable as work, the electrical exergy is given by:

19 $$\:E{x}_{el}=IV$$

The overall exergy efficiency of the hybrid system is determined by combining the thermal and electrical exergy outputs relative to the solar exergy input:

20 $$\:{{\upeta\:}}_{\mathrm{E}\mathrm{x}}=\frac{\mathrm{E}{\mathrm{x}}_{\mathrm{th\:}}+\mathrm{E}{\mathrm{x}}_{\mathrm{e}\mathrm{l}}}{\mathrm{E}{\mathrm{x}}_{\mathrm{in\:}}}$$

Where the incoming solar exergy is computed using the Petela–Landsberg equation^34^:

21 $$\:E{x}_{\mathrm{in\:}}={A}_{pv}G\left[1-\frac{4}{3}\left(\frac{{T}_{\mathrm{amb\:}}}{{T}_{\mathrm{sun\:}}}\right)+\frac{1}{3}{\left(\frac{{T}_{\mathrm{amb\:}}}{{T}_{\mathrm{sun\:}}}\right)}^{4}\right]$$

Exergy utilization has the potential to enhance the solar dryer’s efficacy significantly. Consequently, the waste exergy ratio (WER) is incorporated into his analysis of the renewable energy approach for sustainable growth. The WER is illustrated in Eq. (8)^35,36^:

22 $$\:WER=\frac{E{x}_{\mathrm{in\:}}-\left(E{x}_{\mathrm{th\:}}+E{x}_{el}\right)}{E{x}_{\mathrm{in\:}}}$$

### Drying evaluation

**Fig. 6:**
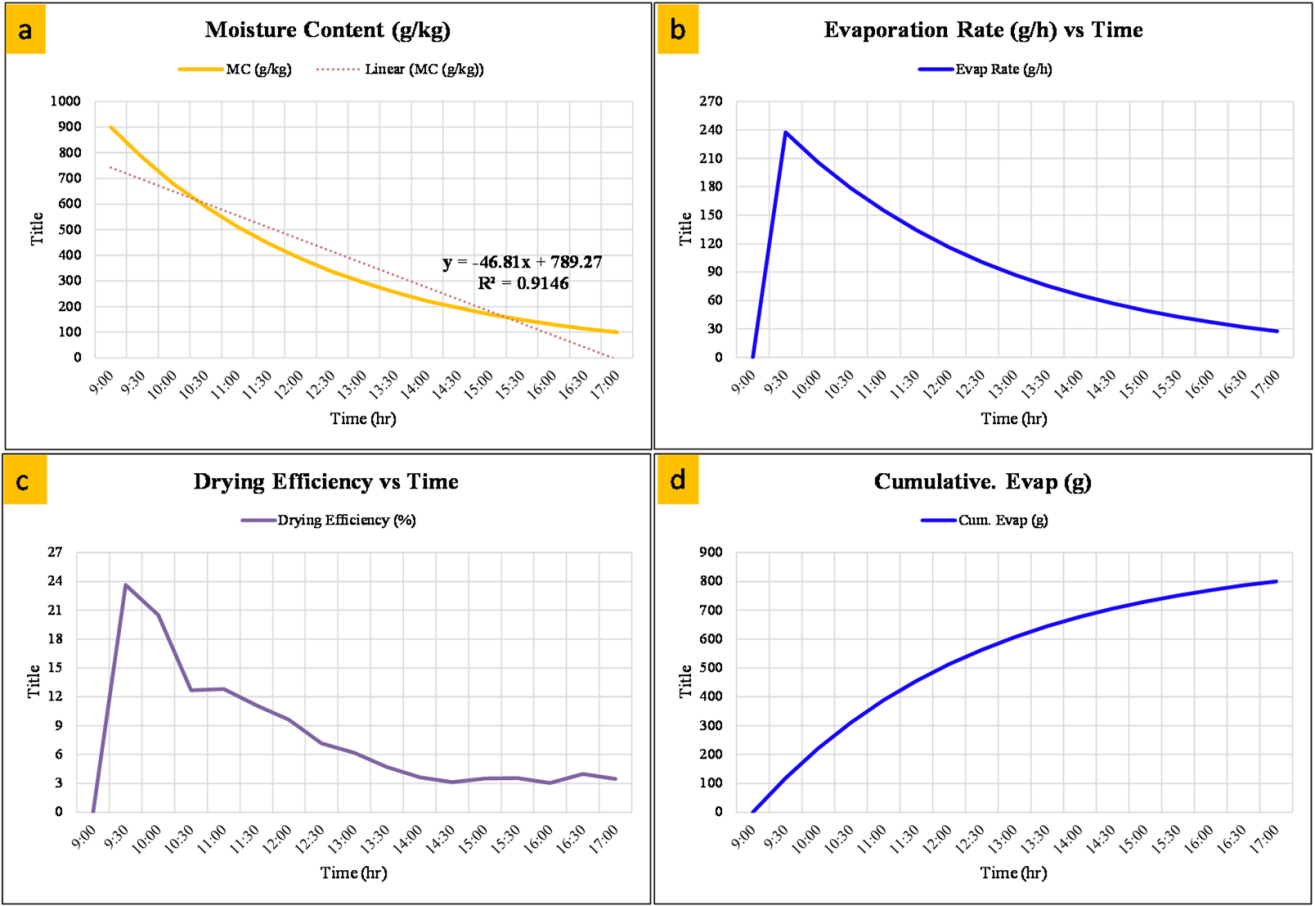
Drying characteristics of tomato: (**a**) variation of moisture content (g/kg) with drying time, (**b**) evaporation rate (g/h), (**c**) drying efficiency (%), and (**d**) cumulative water evaporation (**g**) with time.

The drying process was carried out at variable temperatures between 30 and 53 °C, depending on the weather conditions. Figure 6 shows the drying behavior of 1 kg of fresh tomatoes at an average temperature of approximately 43 °C, displaying variations in moisture content, evaporation rate, drying efficiency, and cumulative water removal over time. The initial moisture content of the tomatoes was high, exceeding 900 g/kg (on a wet basis), and gradually decreased throughout the 8-hour drying period, reaching approximately 100 g/kg by the end of the process, as shown in Fig. 6a. The evaporation rate showed a sharp increase initially, peaking at approximately 240 g/h during the first 30 min due to the availability of free surface water and the high humidity gradient between the product and the drying air. Thereafter, the rate declined steadily as internal diffusion became the dominant mechanism, highlighting the shift from surface evaporation to moisture migration from deeper layers, as shown in Fig. 6b. Drying efficiency followed a similar trend, peaking at approximately 24% in the early stage when latent heat utilization was most efficient, before gradually declining as less water remained to evaporate with continued heat input, as shown in Fig. 6c. The cumulative evaporation curve confirmed that approximately 800 g of water was successfully removed over the drying period, corresponding to a significant reduction in tomato mass, as shown in Fig. 6d.

### TEC and condensation evaluation

**Fig. 7:**
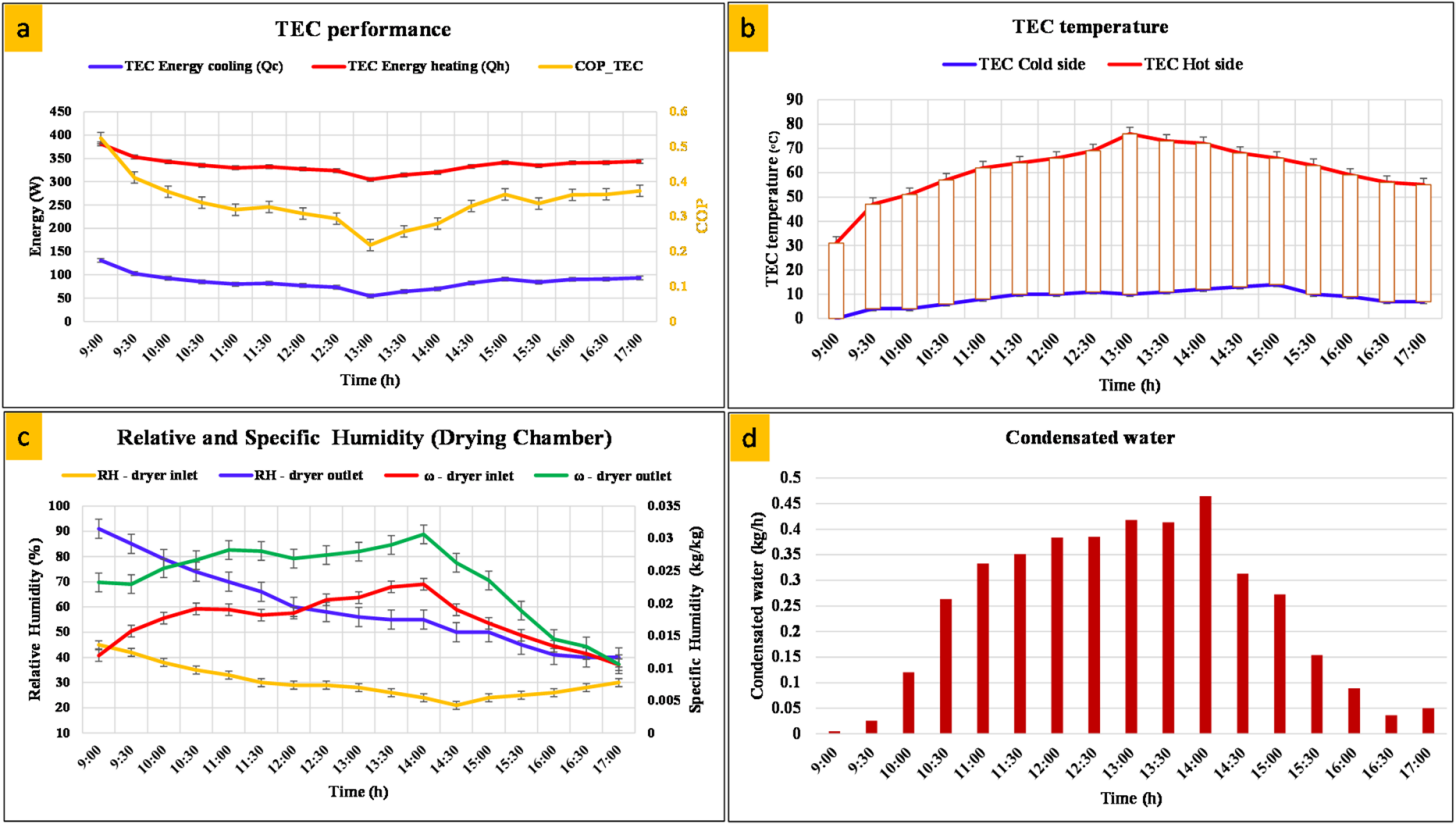
Performance evaluation of the TEC: (**a**) TEC cooling and heating energy with COP, (**b**) temperature variation of TEC hot and cold sides, (**c**) relative and specific humidity variation, and (**d**) time-dependent condensed water yield.

Figure 7 illustrates the thermodynamic performance of the thermoelectric cooler. As shown in Fig. 7a, the heating power (Q_h_) was consistently higher than the cooling power (Q_c_), with values ranging from 250 to 350 W and 50–100 W, respectively. This discrepancy is attributed to the Joule thermal effect and the inherent conversion inefficiency of thermoelectric coolers, where a portion of the supplied electrical energy is dissipated as heat. The COP showed values ranging from 0.2 to 0.5, with higher COPs observed during mid-morning and afternoon when the cooling load was relatively stable, confirming that the operation of the thermoelectric cooler is highly dependent on the input current and ambient thermal conditions. The corresponding temperatures of the hot and cold sides of the thermoelectric cooler, shown in Fig. 7b, further substantiate this trend. The hot side temperature rose steadily from approximately 40 °C in the morning to a maximum of 75 °C around 13:00, due to the combined effect of solar intensity and internal resistive heating, before gradually decreasing in the late afternoon. Figure 7c shows the evolution of relative humidity (RH) and specific humidity (ω) in the drying chamber. At the inlet, the relative humidity gradually decreased from approximately 50% to less than 20% as the drying air continued to be heated by the PV/T collector, while the outlet relative humidity values ​​were consistently higher, indicating moisture absorption from the tomato samples. The outlet specific humidity peaked at approximately 0.032 kg/kg during the afternoon hours, reflecting the maximum evaporation rate when the drying air was at its hottest and capable of holding more water vapor. Finally, the condensed water volume, shown in Fig. 7d, followed a bell curve, with the highest production (~ 0.45 kg/h) occurring between 12:00 and 14:00, coinciding with the peak solar radiation and the maximum temperature difference between hot and cold air across the TEC. It is worth mentioning that adding phase change materials reinforces the cooling strategies in TEC for thermal management applications as discussed by^37^.

## Conclusions

The experimental and analytical evaluation of a hybrid PV/solar dryer with integrated water recovery shows its strong potential for sustainable agricultural processing. Drying tests conducted on 1 kg of tomato slices revealed that the system effectively reduced the moisture content from its initial level to the safe storage level during the designed drying period, achieving maximum thermal efficiency of 53.29%, electrical efficiency of 17%, and overall efficiency of over 70% depending on the weather conditions. The water recovery unit successfully condensed an average of 3.9 L of clean water during an 8-hour operation under typical solar radiation and humidity conditions. Moreover, integrating photovoltaic and thermal energy pathways minimized energy losses and enhanced system performance compared to conventional dryers. The results confirm that the proposed system ensures high-quality dried products, contributes to energy conservation, reduces dependence on fossil fuel-based dryers, and improves water security. Therefore, this work provides a scalable and environmentally efficient solution for rural communities and food industries, aligning with the global goals of sustainable development, circular economy, and resource optimization.

## Data Availability

Any required data will be made available upon request from the corresponding author.
